# How I do it: minimally invasive surgical decompression for lumbosacral extraforaminal stenosis (Far-Out Syndrome)

**DOI:** 10.1007/s00701-025-06553-0

**Published:** 2025-05-14

**Authors:** Subum Lee, Sunghyun Kwon, Seok-Joon Kim, Jun-Hyeok Song

**Affiliations:** 1 Department of Neurosurgery, Korea University Anam Hospital, Korea University College of Medicine, Seoul, Republic of Korea; 2 Department of Neurosurgery, Korea University Ansan Hospital, Korea University College of Medicine, Ansan-Si, Republic of Korea; 3 Department of Neurosurgery, Jounachim Hospital, 144, Gyeongchun-Ro, Guri-Si, 11924 Gyeonggi-Do Republic of Korea

**Keywords:** Bertolotti syndrome, Far-out syndrome, Lumbosacral transitional vertebra, Minimally invasive surgery, Paraspinal approach, Pseudoarticulation

## Abstract

**Background:**

Accurate diagnosis of extraforaminal entrapment of the L5 nerve root, commonly referred to as "far-out syndrome," is challenging due to its unique anatomical characteristics, which differ from those of other lumbar regions. Inadequate decompression may lead to poor outcomes.

**Method:**

A minimally invasive paraspinal approach utilizing a tubular retractor was used to decompress extraforaminal entrapment of the L5 nerve root. Procedures and discussions regarding indications, diagnoses, surgical endpoints, and ways to avoid complications were described.

**Conclusion:**

Adequate decompression requires sufficient resection of the L5 lower vertebral body bony spur, transverse process, and sacral ala as a ventral margin.

**Supplementary Information:**

The online version contains supplementary material available at 10.1007/s00701-025-06553-0.

## Relevant surgical anatomy

Pseudoarticulation or fusion between the transverse process (TP) of L5 and the sacral ala can lead to extraforaminal entrapment of the L5 nerve root [10]. When anteroposterior (AP) X-ray images are reviewed, the gap between the TP and the sacral ala on the affected side is markedly narrower than that on the opposite side. Compared to extraforaminal nerve compression at the above levels, the pathophysiology of L5 extraforaminal nerve compression is more complex, involving multiple structures surrounding the narrow lumbosacral tunnel, which may compress the nerve as it passes through [6, 7]. The L5 TP, the sacral ala, and osteophytes from the L5 vertebral body together pinch the L5 root in a vertical direction from both superior and inferior aspects. Consequently, unilateral claudication usually occurs within the first 5 min of standing or walking. Since the dorsal root ganglion is directly compressed, this condition presents with severe cramping pain in the ipsilateral L5-specific dermatome over shorter distances compared to lateral recess stenosis, where the traversing root is compressed.

**Fig. 1:**
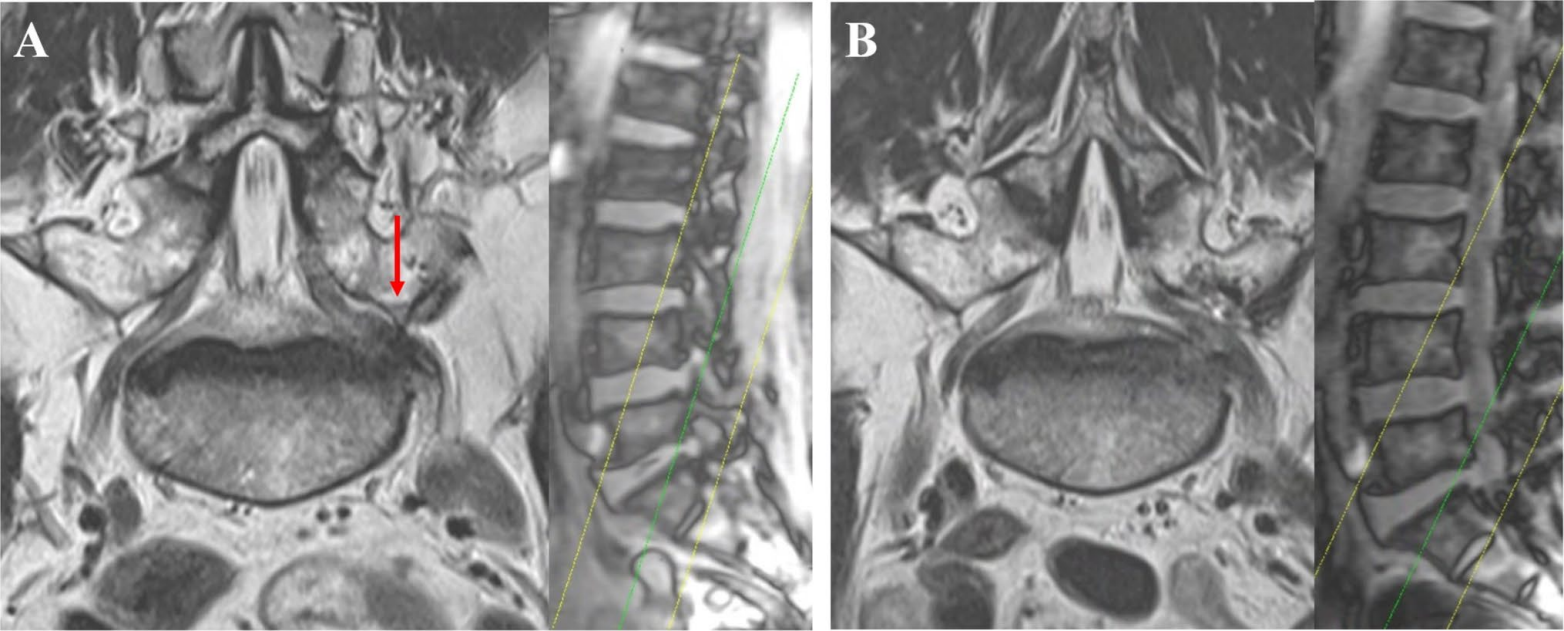
Pre- and postoperative oblique coronal MR images of left L5-S1 extraforaminal stenosis. In each figure, the sagittal scout view on the right illustrates the slicing trajectory through the area of interest along the green line. **A** Preoperative MR image in the oblique coronal plane, with the red arrow indicating compression of the nerve root by the L5 TP. **B** Postoperative MR image showing the decompressed left L5 nerve root in the extraforaminal area

Conventional axial and sagittal magnetic resonance (MR) images may result in incorrect diagnoses [2, 8]. Hence, we routinely perform oblique coronal MR imaging to detect suspected cases of L5 extraforaminal entrapment. The oblique coronal MR imaging effectively identifies the surgical anatomy required for the decompression of the L5 root (Fig. 1).

## Description of the technique

**Fig. 2:**
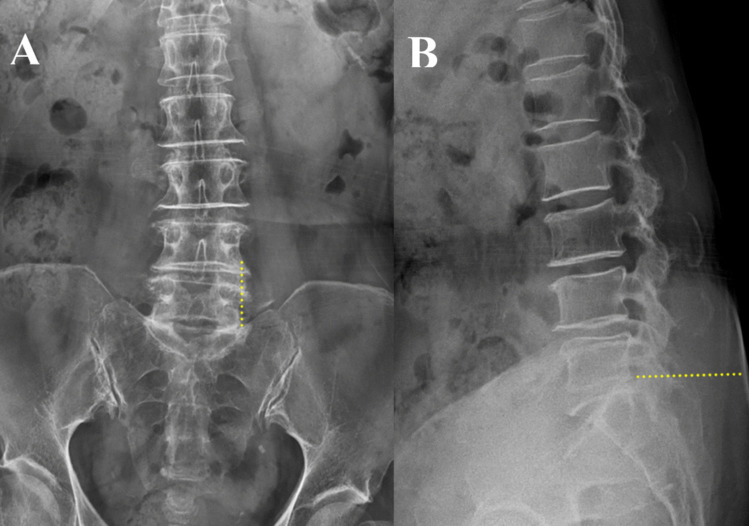
AP and lateral view of x-ray images. **A** AP view where the yellow dotted line represents the skin incision line aligning with the lateral margins of the pedicles. The caudally prominent tip of the left L5 TP, identified on the oblique coronal MR image, is also visible on the AP X-ray. **B** In the lateral view, the skin incision should be made at the right end of the dotted line, oriented toward the posteroinferior corner of the L5 vertebral body

The skin incision is strategically planned based on fluoroscopic AP views, aligning with the lateral margin of the pedicle and extending vertically toward the posteroinferior corner of the L5 vertebral body on lateral views (Fig. 2). An incision length of approximately 28 mm is optimal. The Metrx tube, 22 mm in size, is employed in the incision for the procedure.

**Fig. 3:**
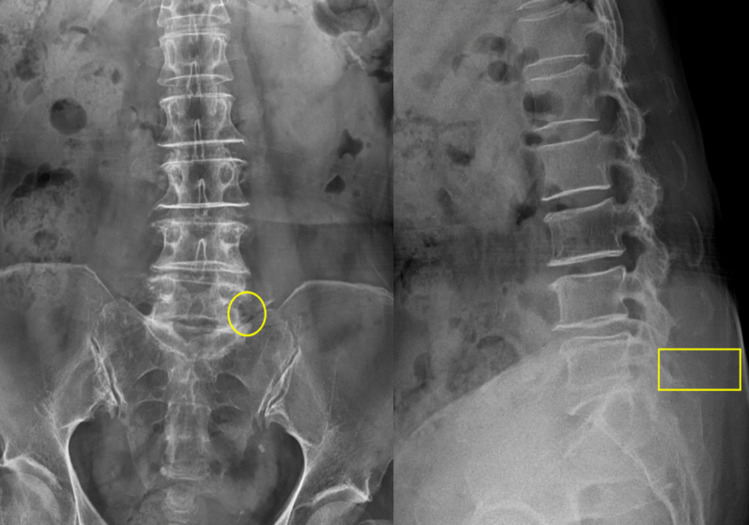
AP and lateral x-ray images. **A** AP view showing the yellow circle representing the position of an ideally placed Metrx tube with the inferolateral margin of the pedicle, the TP, the SAP, and the sacral ala are all included within its field. **B** In the lateral view, the Metrx tube should be inserted in alignment with the yellow box, ensuring that both the lower portion of L5 and the superior corner of S1 are visible within the tube’s field of view

Following the insertion of the Metrx tube, an ideal confirming fluoroscopic AP image should have the following structures within the tube’s circle: a quarter of the pedicle's inferolateral margin, the TP, the superior articular process (SAP), and the ala. On lateral images, it is best recommended that the Metrx tube’s circle encompass the lower half of the L5 pedicle and a slight portion of the S1 posterior-superior corner (Fig. 3).

Except in cases of lytic spondylolisthesis, the TP and the sacral ala are nearly always in direct contact (‘kissing’). In most cases, the TP is typically thicker than 1 cm, and it is reasonable to assume that the TP is at least 1.5 cm thick. Given the significant anatomical distortions due to degenerative changes, frequent fluoroscopic verification is advised during surgery when any doubt arises. The uneven surface where the tube docks may trap substantial soft tissue, which should be thoroughly removed.

**Fig. 4:**
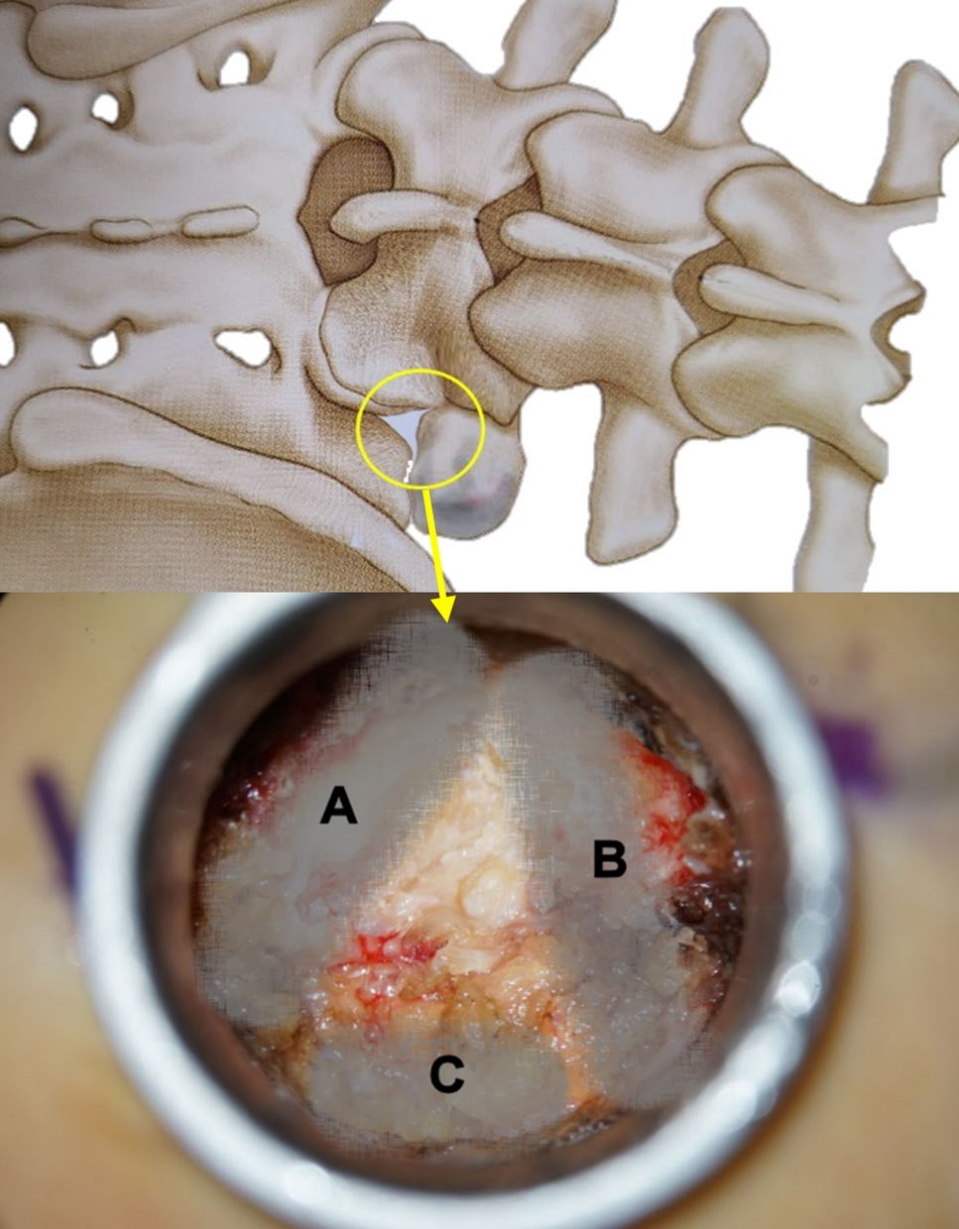
Schematic illustration and intraoperative photograph showing three key bony landmarks: **A**, **B**, and **C**. Landmark **A** corresponds to the consecutive layers of the isthmus, SAP, and vertebral body; **B** mainly represents the TP; and **C** corresponds to the sacral ala

The starting point of drilling is at the lateral margin of the isthmus. It is helpful to visualize a triangle formed by three bony landmarks, A, B, and C (Fig. 4), within the Metrx’s surgical field. The landmarks forming this triangle are drilled extensively with a 5 mm burr to create a triangular entrance. Landmark A has two distinct layers: The dorsal layer corresponds to the isthmus, and drilling through it exposes the SAP below, leading toward the vertebral body and guiding access to the disc. Landmark B is mainly the TP. The complete removal of the lower portion of the TP eliminates the superior compressive pathology caused by disc height loss and provides additional room for surgery around the L5 root. Landmark C involves drilling the sacral ala to remove the inferior compressive pathology.

Gradual drilling of A, B, and C toward the ventral side is advised to preserve the ligaments for root protection. Upon removing the ligaments, the L5 root is exposed (video clip). During this process, bleeding from the radicular artery may occur, which can be controlled with bipolar cautery. Identification of the root margin and inspection for disc bulging or ventral bony spurs are performed with a right-angled blunt hook. These protruding structures are then carefully drilled and extracted using pituitary forceps (video clip). The prior removal of the lower part of the TP provides a safe room, allowing for bone work without root injuries. Finally, bone in direct contact with the root is drilled down to an approximate thickness of 1 mm, resembling an eggshell, and then safely removed via curettage and pituitary forceps for decompression.

This technique highlights the importance of meticulous planning and execution in the surgical management of far-out syndrome, ensuring patient safety and optimal outcomes.

## Indications

I. Unilateral radiating leg pain in the L5 dermatome that is refractory to conservative management.
II. Temporary relief of unilateral radicular pain after a selective L5 root block procedure.
III. MRI evidence of extraforaminal compression of L5 with the kissing of the TP and sacral ala for more accurate expression.

## Limitations

In most cases, patients present with unilateral rather than bilateral symptoms. This is primarily due to the consensus that bilateral symptoms are commonly associated with lytic spondylolisthesis, where fusion and fixation are preferred treatment options. Instead of bilateral extraforaminal decompression, pedicle screw fixation and fusion are typically more practical and effective. Consequently, extraforaminal decompression has a limited scope of application and should be carefully considered based on its indications.

## How to avoid complications

Decompressive surgery is performed with the patient in a prone-flexion position. However, in the standing position, lumbar extension reduces the distance between the L5 TP and the sacral ala. This narrowing leads to vertical compression of the L5 nerve root. Therefore, even if the L5 nerve root appears adequately decompressed within the surgical field, additional bone work is required to further widen the space between the TP and the sacral ala in the vertical direction. Additionally, since the nerve root travels from an outward to a ventral direction, it is essential to accurately identify and remove any ventral bony spurs of the vertebral body, if present [9].

## Specific information for the patients regarding clinical presentations

Patients with lumbosacral extraforaminal stenosis commonly experience low back pain, unilateral sciatic pain, weakness of the extensor hallucis longus muscle, hypoesthesia in the corresponding dermatome, and neurogenic claudication [6, 5, 4]. Claudication typically develops unilaterally within the first five minutes of standing or walking. Bilateral claudication or cauda equina syndrome is rare unless concomitant lumbar intraspinal stenosis is present [1]. Mild scoliosis is commonly associated, with the convexity of the curve tending toward the symptomatic side [6]. As a result, AP X-ray typically shows a narrower gap between the L5 TP and the sacral ala on the symptomatic side than on the contralateral side. Electrophysiological evaluation may play a decisive role in accurately diagnosing the lesion [3]. Radiculopathy, peripheral mononeuropathy, and plexopathy may present similarly, and other conditions such as intraspinal stenosis, disc herniation, and facet joint disease should be carefully considered in the differential diagnosis [1].

## Supplementary Information

Below is the link to the electronic supplementary material.

Supplementary file1 (MP4 99.0 MB)

## Data Availability

No datasets were generated or analysed during the current study.
